# Adaptation of Brown Adipose Tissue in Response to Chronic Exposure to the Environmental Pollutant 1,1-Dichloro-2,2-bis(p-chlorophenyl) Ethylene (DDE) and/or a High-Fat Diet in Male Wistar Rats

**DOI:** 10.3390/nu16162616

**Published:** 2024-08-08

**Authors:** Vincenzo Migliaccio, Ilaria Di Gregorio, Serena Penna, Giuliana Panico, Assunta Lombardi, Lillà Lionetti

**Affiliations:** 1 Department of Chemistry and Biology “A. Zambelli”, University of Salerno, Via Giovanni Paolo II, 132, 84084 Fisciano, Italy; vmigliaccio@unisa.it (V.M.); idigregorio@unisa.it (I.D.G.); spenna@unisa.it (S.P.); 2 Department of Biology, University of Naples Federico II, Complesso Monte Sant’Angelo Via Cinthia 26, 80126 Napoli, Italy; giuliana.panico@unina.it

**Keywords:** metabolism, pesticides, mitochondria, oxidative stress, hyperlipidic diet

## Abstract

Brown adipose tissue (BAT) participates in thermogenesis and energy homeostasis. Studies on factors capable of influencing BAT function, such as a high-fat diet (HFD) or exposure to environmental pollutants, could be useful for finding metabolic targets for maintaining energy homeostasis. We evaluated the effect of chronic exposure to dichlorodiphenyldichloroethylene (DDE), the major metabolite of dichlorodiphenyltrichloroethane (DDT), and/or a HFD on BAT morphology, mitochondrial mass, dynamics, and oxidative stress in rats. To this end, male Wistar rats were treated for 4 weeks with a standard diet, or a HFD alone, or together with DDE. An increase in paucilocular adipocytes and the lipid droplet size were observed in HFD-treated rats, which was associated with a reduction in mitochondrial mass and in mitochondrial fragmentation, as well as with increased oxidative stress and upregulation of the superoxide dismutase-2. DDE administration mimics most of the effects induced by a HFD on BAT, and it aggravates the increase in the lipid droplet size when administered together with a HFD. Considering the known role of oxidative stress in altering BAT functionality, it could underlie the ability of both DDE and a HFD to induce similar metabolic adaptations in BAT, leading to reduced tissue thermogenesis, which can result in a predisposition to the onset of energy homeostasis disorders.

## 1. Introduction

Brown adipose tissue (BAT) participates in thermogenesis. Brown adipocytes contain a lot of lipid droplets and a large number of mitochondria expressing uncoupling protein-1 (UCP1) [1,2]. The tissue burns lipids and carbohydrates to produce heat, by dissipating the mitochondrial proton-motive force, generated by the activity of the respiratory chain, as heat [1,3], through the activation of UCP1. To maintain and prolong thermogenesis, the tissue essentially uses lipids stored in droplets and substrates from the circulation (i.e., lipids, glucose, and lactate). Therefore, BAT activation, by promoting changes in the serum levels of triglycerides and glucose, plays an important role in metabolic and energetic homeostasis [1,4,5].

Interestingly, it has been shown that, in adults human, BAT activity positively correlates with the resting metabolic rate, while it is negatively associated with body mass index, fat mass, and the occurrence of dysmetabolic diseases, such as obesity, diabetes, and insulin resistance [6]. In addition, BAT transplantation has been shown to reverse obesity in rodents [7,8,9]. Thus, the individuation of factors able to influence BAT functionality and mitochondrial thermogenesis is important to clarify the etiology of some dysmetabolic diseases, which in Western countries are becoming even more frequent and are associated with unhealthy lifestyles.

A chronic high-fat diet (HFD) is one of the factors that is able to influence BAT functionality. The first pieces of evidence on the relationship between a HFD and BAT physiology indicated diet-induced thermogenesis as a physiological defense mechanism against diet-induced obesity [10]. However, although the short-term administration of a HFD is associated with increased mitochondrial activity and thermogenic capacity of BAT [11], a plethora of evidence has shown that persistent excess dietary fat leads to systemic metabolic disturbance. Indeed, the chronic administration of a HFD depletes mitochondrial functionality and promotes uncontrolled mitochondrial production of ROS in highly oxidative tissues, including BAT [12,13,14]. Furthermore, the metabolic response activated by a HFD in BAT is accompanied by changes in mitochondrial dynamics, characterized by reduced fragmentation and reduced mitochondrial content [12]. Moreover, chronic HFD intake caused BAT dysfunction and whitening, with defective non-shivering thermogenesis, the upregulation of inflammasome activation, and the presence of endoplasmic reticulum stress markers [15].

Another factor that has emerged in recent years as capable of influencing BAT functionality is the exposure to environmental pollutants [16,17,18,19,20,21]. The current literature supports the hypothesis that different types of environmental pollutants could affect BAT differently [16]. Chemicals, such as perfluorooctane sulfonate (PFOS) and perfluorooctanoic acid (PFOA), elicited a reduction in body weight and adipose mass associated with the upregulation of UCP1 and increased oxidative capacity in BAT mitochondria [16,20,22]. On the other hand, air pollutants induced a decrease in UCP1 content and mitochondrial size/number in BAT associated with insulin resistance [16,19]. Moreover, some environmental pollutants, acting as endocrine disruptors (EDCs), such as dichlorodiphenyltrichloroethane (DDT) and its metabolite dichlorodiphenylethylene (DDE), impaired BAT mass and function [16]. Perinatal exposure to DDT in mice impaired BAT thermogenesis and substrate utilization, thus increasing the susceptibility to metabolic syndrome in female adult offspring [23,24].

Considering that in modern Western societies humans are often chronically exposed to both a HFD and environmental pollutants, it would be useful to study how environmental pollutants can influence the functionality of BAT in conditions of simultaneous chronic exposure with a normolipidic or hyperlipidic diet. In recent years, our research group has analyzed the effect of chronic exposure to a non-toxic dose of the environmental pollutant DDE associated with a low-fat or high-fat diet in an experimental animal model of male Wistar rats, focusing on liver and testicular tissues [25]. We chose to use DDE in our experimental design, as it is the most persistent metabolite of DDT. DDT has largely been used as an insecticide to eradicate malaria, typhus, and other diseases carried by phlebotomies and/or mosquitoes, and employed in agriculture. Nowadays, given its toxicity for animal species, DDT use is only allowed in equatorial areas for sanitary aims [26]. However, DTT persists in environmental compartments [27] and, due to its hydrophobic character, it accumulates in animal organs, principally in adipose tissue [28]. In the different environmental compartments, DDT undergoes biotic or abiotic dechlorination processes over time, producing the much more persistent metabolite DDE, which shows a half-life of more than 7 years [29]. DDT and DDE have been shown to interfere with cell physiology, and the number of pieces of evidence on the obesogenic effects exerted by these molecules has increased notably in the last decade [30].

Cellular toxicity can be mediated by oxidative stress; therefore, antioxidant defenses can be useful for the adaptive response of the tissue to external toxic agents. In our previous study, we showed that in the liver of male Wistar rats exposed to DDE and/or a HFD, antioxidant system stimulation and mitochondrial UCP2 upregulation seemed to play a potential protective role in counteracting ROS overproduction, mainly in the presence of the environmental pollutant [31]. Moreover, in testicular tissue, a HFD induced lipotoxicity and altered the oxidative balance. On the other hand, DDE generated higher tissular alterations, with the impairment of spermatogenesis, with a worse redox state when the pesticide exposure was not associated with a HFD [25].

Interestingly, different authors suggest that the accumulation of DDT and DDE in the adipose organ could preserve other tissues through the pro-oxidant and deleterious effects of the pesticides [32]; however, the exposure over time could induce metabolic alterations in the organ in which they are stored. In this context, both DDT and DDE could alter adipose tissue function by affecting cellular organelles, including mitochondria [33].

In the literature, studies concerning the adaptation occurring in BAT due to DDE chronic exposure are very limited. DDE, administered from gestational day 11.5 until postnatal day 5, reduced the core body temperature and the synaptic density in stellate ganglia innervating BAT, but it did not affect lipid storage in the tissue and/or sympathetic innervation in 4-month-old female mice offspring [24]. Other studies investigating the obesogenic effects of organochlorine pesticides, limited their focus relative to the impact of DDE on BAT to the evaluation of mRNA expression of UCP1, the peroxisome proliferator-activated receptor gamma (PPARγ), and UCP3, which remained unaffected [30]. Taking into account previous considerations, in the present research work we considered it interesting to continue our investigation into the effect of chronic exposure to non-toxic doses of DDE administered simultaneously with a standard diet or with a HFD, focusing on BAT, and analyzing mitochondrial impairment and oxidative stress generation in the tissue. In addition, we focused our attention both on the whole tissue homogenate and on isolated mitochondria.

Our experimental model allows for discrimination if DDE and a HFD have similar, synergic, or contrasting effects. Furthermore, it also allows us to distinguish whether exposure to DDE has different effects on BAT depending on the diet it is associated with, whether a normal or high-fat diet.

This aspect gains significance in view of the evidence that, in modern society, traditional dietary patterns are replaced by diets rich in saturated fats, thus humans are currently exposed to pollutants and a HFD, with consequent increased risk of related metabolic diseases. Studies on the response of tissues to these environmental stimuli can be useful in understanding the adaptive response required to maintain a healthy condition and, therefore, to find cellular and tissular targets for the prevention and therapy related to environmental stimuli-induced metabolic diseases.

## 2. Materials and Methods

### 2.1. Chemicals and Reagents

All the chemicals were purchased from Merck (Sigma St. Louis, MO, USA) unless otherwise specified.

### 2.2. Animals

To perform the analyses described in this paper, the animals used are the same as those reported in our previous work [31]. Briefly, male Wistar rats (Charles River Italia, Como, Italy) were used and housed at 24 °C, one per cage, in a temperature-controlled room, under a 12:12 h light–dark cycle. The rats had free access to food and water.

In particular, 4 experimental groups, consisting of 8 animals in each one, were obtained: the control group (ND) was fed with a standard control diet (standard control diet PF1915, HTD.06416 Harlan Laboratories, Milano, Italy, 15.47 KJ/g, 10% fat, 20% proteins, 70% carbohydrates, for 4 weeks). The animals received a daily oral administration of 200 μL of corn buffer oil. Another group of animals (ND+DDE) was fed with the above standard diet and received a daily oral administration of DDE (10 mg/Kg body weight) dissolved in 200 μL of corn oil.

The high-fat diet-treated group (HFD) was fed ad libitum with a fat-enriched diet (PF1916, HTD.06415 Harlan Laboratories, 19.23 KJ/g, 45% fat, 20% proteins, 35% carbohydrates); the animals received a daily oral administration of 200 μL of corn oil. Another group of animals (HFD+DDE) was fed with the above HFD and received a daily oral administration of DDE (10 mg/Kg body weight) dissolved in 200 μL of corn oil.

The dose of DDE administered has been reported as not being able to influence the physical development and sexual maturation of male pubertal and older rats [34,35]. The characterization of the animal model in terms of body weight gain, triglyceride serum levels, cholesterol serum levels, and fat pad have been reported in previous studies [31].

This study was performed in accordance with the recommendations in the EU Directive 2010/63/EU on the care and use of laboratory animals. The protocol was approved by the Committee on the Ethics of Animal Experiments at the University of Naples Federico II (Permit Number: 2012/0024690).

At the end of the treatment period, the rats were anaesthetized with Zoletil (40 mg/Kg body weight) and euthanized by decapitation.

### 2.3. Adipose Tissue Weight, Processing, and Analysis

Interscapular BAT was dissected, weighed, and immediately processed for histological analyses, or frozen in liquid nitrogen. Tissue fragments were fixed in Bouin’s fixative solution O.N. at room temperature, dehydrated in ethanol, embedded in paraffin, and cut using a microtome at 5 μm for slide preparation. The morphology was studied by staining sections with a classical protocol of hematoxylin and eosin.

### 2.4. Oxidative Stress Measurement

The BAT ROS levels were measured following the ROS-induced conversion of 2′,7′-dichlorodihydrofluorescin diacetate (DCFH-DA, Sigma St. Louis, MO, USA nonfluorescent compound) in dichlorofluorescein (DCF, fluorescent compound), as reported by Driver et al. [36]. The tissue was homogenized using polytron in 5 vol of phosphate buffer 0.1 M, pH 7.4, and then diluted in Locke’s buffer (154 mM NaCl, 5.6 mM KCl, 3.6 mM NaHCO_3_, 2 mM CaCl_2_, 10 mM glucose, 5 mM Hepes). Moreover, 25 µg BAT homogenate proteins in 200 μL of Locke’s buffer were incubated for 15 min with 10 µM DCFH-DA. Then, FeCl_3_ was added to reach the final concentration of 100 µM; the mixture was incubated for 30 min. A Tecan Infinite 200 pro microplate reader was used to detect the conversion of DCFH-DA into the fluorescent product DCF (λex 485 nm, λem 530 nm). The conversion of DCFH into DCF in the absence of the BAT homogenate was used as background fluorescence. A standard curve of DCF was used to calculate the pmol DCF formed. The BAT free radical levels were detected in tissue homogenate using a spectrofluorimetric assay.

Oxidative damage to the lipids was evaluated spectrophotometrically by detecting the thiobarbituric acid reactive substance (TBARS) levels, as reported by De Leon and Borger [37].

### 2.5. Mitochondrial Isolation

An interscapular BAT mitochondrial-enriched fraction was obtained, as described by Lombardi et al. [38]. Briefly, the BAT was deprived of all visible WAT contamination, immersed in isolation buffer [220 mM mannitol, 70 mM sucrose, 20 mM Tris-HCl, 1 mM EDTA, 5 mM EGTA, and 1% fatty acid-free bovine serum albumin (BSA)], and homogenized in a Potter–Elvehjem homogenizer. An aliquot of the homogenate was collected for biochemical analyses. The remaining homogenate was centrifuged at 8000× *g* for 10 min at 4 °C. Floating fat was removed. The pellet was resuspended in the supernatant and centrifuged at 700× *g* for 10 min. The resulting supernatant was centrifugated at 3000× *g* to obtain a mitochondrial pellet, which was washed twice. Enzymatic and electron microscopy characterization has shown that our isolation procedure (centrifugation at 3000× *g* for 10 min) results in a cellular fraction, which is essentially made up of pure mitochondria [39,40,41].

### 2.6. Cytochrome Oxidase Activity

The cytochrome oxidase activity was assessed in terms of BAT homogenate and isolated mitochondria. The procedure used, described by Lombardi et al. 2015, was conducted at 25 °C using a Clark-type oxygen electrode [38].

### 2.7. BAT Total Protein Extraction and Western Blotting

Tissue fragments were homogenized in ice-cold RIPA buffer (150 mM NaCl, 1.0% Triton X-100, 0.5% sodium deoxycholate, 0.1% SDS, 50 mM Tris, pH 8.0), supplemented with protease cocktail and phosphatase inhibitors (Sigma Aldrich, Sigma St. Louis, MO, USA). The homogenates were left on ice for 1 h, vortexed every 10 min to promote protein extraction. The lysate was ultracentrifuged at 17,000× *g* for 20 min at 4 °C. Both the floating fat and pellet were discarded, and the supernatants were used for protein quantification and analyses.

For the Western blot analysis, the following primary antibodies were used: Mfn2 (Santacruz Biotechnology, Heidelberg, Germany, sc-100560, 1:300); Drp1 (Santacruz Biotechnology, sc-271583, 1:1000); Plin1 (Abcam, Milano, Italy, ab 3526, 1:1000); VDAC (Santacruz Biotechnology, sc-390996, 1:1000); UCP1 (Abcam, ab 10983, 1:1000); PGC1α (Cell Signaling Tech, Milano, Italy, #2178S, 1:1000); SOD2 (Cell Signaling Tech, D3 × 8F, 1:2000); and Tubulin (Cell Signaling Tech, #2146). Appropriate secondary anti-mouse (Goat Anti-Mouse IgG-HRP Conjugated, 1:2000–1,706,516 Biorad, Milano, Italy) and anti-rabbit (Goat Anti-Rabbit IgG-HRP Conjugated, 1:2000–1,706,515 Biorad) antibodies were used.

The protein representation was quantified by densitometry using Image J software 1.54j and normalized based on loading control, namely in regard to Tubulin. Data were represented as the amount of change vs. control group (ND), which was set at 1.

### 2.8. Immunohistochemical Stain

Immunohistochemical reactions were performed by using the Novolink polymer detection kit system (Leica Biosystem, Milano, Italy). The slides were processed as previously described [31]. The sections were incubated O.N. at 4 °C with the following antibodies: Mfn2, Santacruz Biotechnology, sc-100560, 1:300; Drp1, Santacruz Biotechnology, sc-271583, 1:500; Plin1, Abcam ab 3526, 1:1000; UCP, Abcam, ab 10983, 1:300. Images were obtained using a Zeiss Axioskop microscope, Oberkochen, Germany, fitted with a TV camera.

### 2.9. Statistical Analyses

Statistical analyses were performed using GraphPad Prism software 8.0.2.263. After checking the normal distribution of the data using the Shapiro–Wilk test, two-way ANOVA analysis, followed by Tukey’s post-test were applied. The data were considered statistically different when *p* < 0.05.

## 3. Results

### 3.1. Effect of DDE Treatments and/or a HFD on BAT Morphology

**Figure 1:**
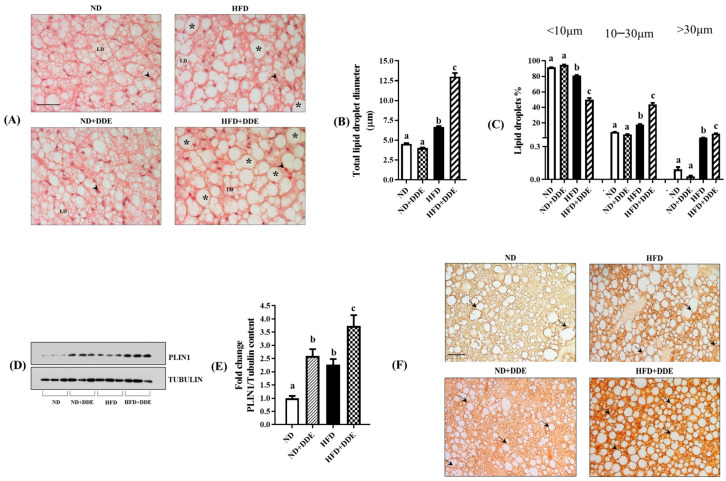
Effects of DDE and/or HFD on BAT morphology, lipid droplet size, and perilipin 1 level. (**A**) BAT sections stained with hematoxylin and eosin. ND and ND+DDE groups showed a multilocular distribution of fatty depots in brown adipocytes. Both lipid droplets (LD) and nuclei (arrowhead) are evidenced in histological images. In HFD and HFD+DDE groups, a change in lipid droplet size was detected (the symbol * indicates paucilocular adipocytes). Magnification used: 20×. Scale bar applied (in ND): 50 μm. (**B**) Total lipid droplet diameter, and (**C**) the percentage of small (diameter < 10 μm) medium (diameter between 10 and 30 μm), and large (diameter > 30 μm) lipid droplets. (**D**) Representative image of PLIN-1 Western blot is shown, and Tubulin has been used as the loading control. (**E**) Histogram represents the quantification of Western blot data. Data were normalized to the values obtained for the ND group, set as 1, and were graphically represented as mean ± SEM of 6 animals per group. Two-way ANOVA analysis, followed by Tukey’s post-test were applied. Bars labeled with different letters are significantly different (*p* < 0.05). (**F**) PLIN1 immunohistochemistry detected in BAT sections. Immunostaining was evidenced around droplets and in cell cytosol (black arrows). Magnification used: 20×. Scale bar applied (in ND): 50 μm. Two-way ANOVA analysis results: lipid droplet mean size; effect of the diet (*p* < 0.0001); effect of DDE (*p* < 0.0001); interaction (*p* < 0.0001). Small Lipid droplet percentage: effect of diet (*p* < 0.0001); effect of DDE (*p* < 0.0001); interaction significant (*p* < 0.0001). Medium lipid droplet percentage: effect of diet (*p* < 0.0001); effect of DDE (*p* < 0.0001); interaction (*p* < 0.0001). Large lipid droplets: effect of diet (*p* < 0.0001); effect of DDE (*p* = 0.0009); interaction (*p* = 0.0007). PLIN1 levels: effect of diet (*p* = 0.0002); effect of DDE (*p* < 0.0001); interaction (*p* = 0.8152).

Histological analyses evidenced that a HFD and DDE influenced fatty depots in brown adipocytes, the effect of DDE being dependent on the diet regime. In fact, in HFD and HFD+DDE rats, the parenchyma displayed many adipocytes that contained lipid droplets of an increased size (paucilocular adipocytes) compared to ND, with HFD+DDE showing a more pronounced effect. On the other hand, ND and ND+DDE showed similar parenchyma morphology and fatty depots (Figure 1A).

To quantify the variation in lipid droplet size associated with the treatments performed, we detected the mean diameter and the percentage of droplets showing a diameter <10 μm (named small droplets), between 10 and 30 μm (named medium droplets), and >30 μm (named large droplets).

Our analyses showed a significant increase in the mean droplet diameter in HFD and HFD+DDE groups vs. ND (1.46 and 2.86-fold, respectively). No variation in the mean lipid droplet diameter was observed between the ND and ND+DDE groups (Figure 1B).

In addition, we detected a progressive reduction in the small droplet percentage in the HFD and HFD+DDE groups vs. the ND group (−11% and −46%, respectively) associated with a progressive increase in the percentage of medium droplets (about 2-fold and 5-fold vs. ND, respectively) and large droplets (about 9-fold and 65-fold vs. ND, respectively) (Figure 1C). Interestingly, a HFD and DDE have interactive effects on the droplet size (see Figure 1 legend).

We also detected BAT perilipin 1 (PLIN1) protein levels, a lipid droplet-associated protein that regulates lipid metabolism in adipocytes by serving as a physical barrier for lipase activities [42], and tissue immunolocalization.

Western blotting analyses evidenced a 2.3-fold and 3.7-fold increase in PLIN1 levels in the HFD and HFD+DDE groups vs. ND group, respectively. Similarly, a 2.6-fold increase in protein levels was observed in the ND+DDE group vs. ND group (Figure 1D). In agreement, the immunohistochemical analyses confirmed the Western blotting data, since it revealed an increase in PLIN1 tissue immunoreactivity (arrows) in the HFD and DDE-treated groups vs. ND group (Figure 1E).

### 3.2. Effect of DDE and/or HFD Treatments on BAT Mitochondria

**Figure 2:**
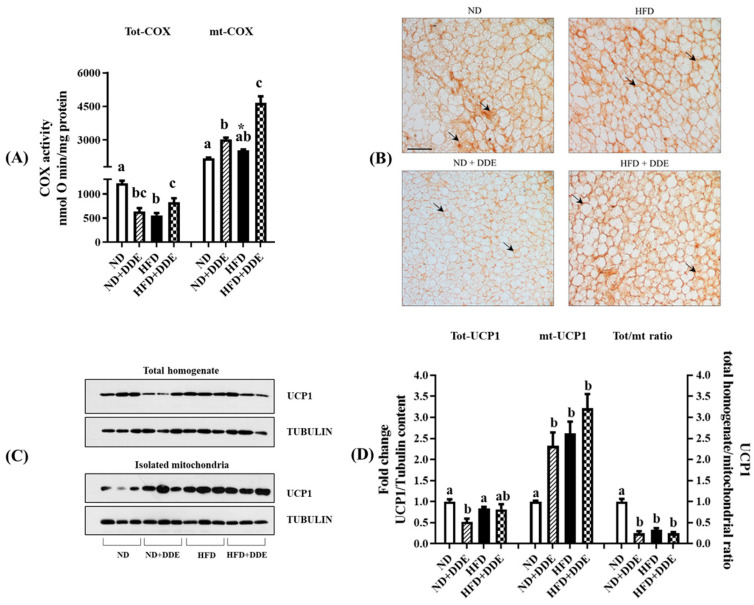
Effect of DDE and/or HFD treatments on UCP1 levels and COX activity. (**A**) COX activity detected both in total homogenates and in isolated mitochondria. The symbol * indicates statistical differences between HFD and ND groups tested by using *t*-test analyses. (**B**) Representative histological section of UCP1 immunostaining. Immunostaining was evidenced by black arrows. (**C**) Western blot of UCP1, detected in BAT lysate and in isolated mitochondria. Tubulin has been used as a loading control. (**D**) Histograms represent the quantification of Western blot data. Data were normalized to the values obtained for the ND group, set as 1, and were graphically represented as mean ± SEM of 6 animals per group. Two-way ANOVA analysis, followed by Tukey’s post-test were applied. Bars labelled with different letters are significantly different (*p* < 0.05). Statistical narrative results evidenced the following effects: UCP1 levels detected in BAT lysate: effect of diet n.s. (*p* = 0.3889); effect of DDE (*p* = 0.0044); interaction (*p* = 0.0082). UCP1 levels detected in isolated mitochondria: effect of diet (*p* = 0.0001); effect of DDE (*p* = 0.0020); the interaction n.s. (*p* = 0.1920). UCP1 homogenate/mitochondria ratio: effect of diet (*p* < 0.0001); effect of DDE (*p* < 0.0001); interaction (*p* < 0.0001). COX activity in BAT homogenates: effect of diet (*p* = 0.0271); effect of DDE (*p* = 0.0014); interaction (*p* < 0.0001). COX activity in BAT mitochondria: effect of diet (*p* < 0.0001); effect of DDE (*p* < 0.0001); interaction (*p* = 0.0004).

To reveal any influence on the tissue and mitochondrial maximal oxidative capacity, we evaluated the cytochrome oxidase complex (COX) activity both in the whole tissue homogenate and in isolated mitochondria (Figure 2A).

The HFD and DDE administration significantly reduced the whole homogenate COX activity (Figure 2A, Tot-COX). Interestingly, the effect of DDE was more pronounced when it was administered to the standard diet-fed mice than to the HFD-fed ones (−48% in ND+DDE vs. ND and −32% in HFD+DDE vs. ND). Moreover, when comparing the HFD and HFD+DDE groups, the COX activity was higher in the second one (1.5-fold).

When detected in isolated mitochondria, the COX activity was significantly stimulated by both the HFD and DDE treatments (Figure 2A, mt-COX); the effect being more pronounced when the two treatments were simultaneously performed. The values observed were increased by about 1.4, 1.2, and 2.14-fold in the ND+DDE, HFD, and HFD+DDE groups, respectively.

To evaluate BAT thermogenic capacity, we detected UCP1 protein content both in the whole tissue and in isolated mitochondria lysates (Figure 2C,D), together with tissue UCP1 immunoreactivity (Figure 2B). The HFD did not affect the total BAT lysate UCP1 levels. DDE administration to HFD fed-rats did not influence the UCP1 protein content, while its administration to the standard diet-fed rats significantly reduced the protein content (−48% vs. ND). Concerning the UCP1 levels detected in mitochondria lysates, we found that all the treatments performed enhanced it. The increases observed were 2.6-fold, 3.2-fold, and 2.3-fold in the HFD, HFD+DDE, and ND+DDE groups vs. ND group, respectively.

We then determined the ratio between the levels of UCP1 detected in the total tissue and those present in isolated mitochondria, which were reduced by 66% in the HFD group, and 75% in both the HFD+DDE and ND+DDE groups compared to the ND group. Given the specific localization of UCP1 in mitochondria, these data suggest that HFD and DDE treatments induce a reduction in tissue mitochondrial content.

**Figure 3:**
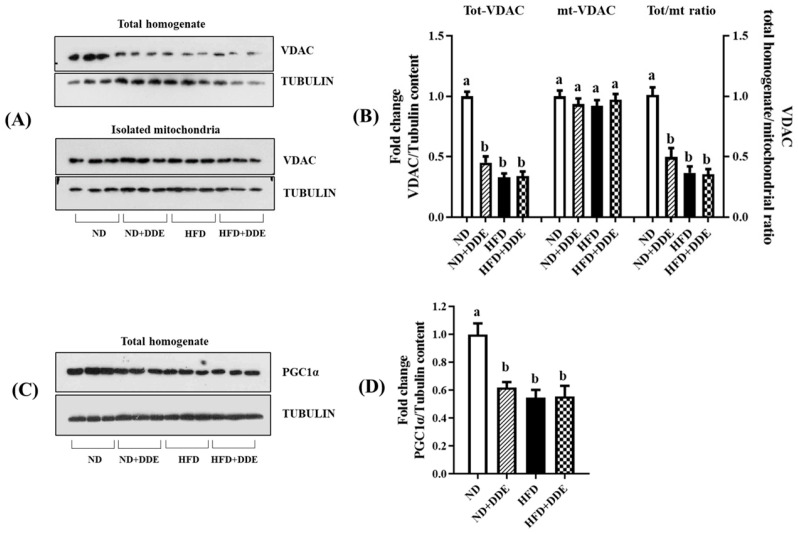
Effect of DDE and/or HFD treatments on VDAC and PGC1α levels. (**A**) Representative Western blot of VDAC detected in BAT lysate and in isolated mitochondria. (**B**) Histograms represent the quantification of Western blot data. (**C**) Representative Western blot of PGC1-α detected in BAT lysate. (**D**) Histograms represent the quantification of Western blot data. Tubulin has been used as a loading control. Data were normalized to the values obtained for the ND group, set as 1, and were graphically represented as mean ± SEM of 6 animals per group. Two-way ANOVA analysis, followed by Tukey’s post-test were applied. Bars labelled with different letters are significantly different (*p* < 0.05). Statistical narrative results evidenced the following effects: VDAC levels detected in BAT lysate: effect of diet (*p* < 0.0001); effect of DDE (*p* < 0.0001); interaction (*p* < 0.0001). VDAC levels detected in isolated mitochondria: effect of diet not significant (n.s.) (*p* = 0.6777); effect of DDE n.s. (*p* = 0.8630); interaction n.s. (*p* = 0.2387). VDAC homogenate/mitochondria ratio: effect of diet (*p* < 0.0001); effect of DDE (*p* = 0.0002); interaction (*p* = 0.0004). PGC1 alfa levels: effect of diet (*p* = 0.0008); effect of DDE (*p* = 0.0099); interaction (*p* = 0.0073).

To sustain this possibility, we detected the levels of the voltage-dependent anion channel (VDAC) protein, a mitochondrial-specific protein located in the outer membrane, and of the peroxisome proliferator-activated receptor gamma coactivator 1α (PGC1-α), a transcriptional co-factor that profoundly influences mitochondrial biogenesis. Concerning VDAC levels were detected in BAT lysate, we reported that all the treatments performed significantly downregulated them, with the values being reduced by 67%, 66%, and 55% in the HFD, HFD+DDE, and ND+DDE groups vs. the ND group, respectively. In isolated mitochondria, no significant changes in VDAC content were observed among the animal groups; therefore, the ratios between VDAC levels detected in the total tissue and that in isolated mitochondria were reduced by 63%, 64%, and 50% in the HFD, HFD+DDE, and ND+DDE groups vs. the ND group (Figure 3A,B).

Referring to PGC1-α, a master regulator of mitochondrial biogenesis, both the HFD and DDE treatments affect them, with the values being reduced by 45%, 44%, and 38% in the HFD, HFD+DDE, and ND+DDE groups vs. the ND group, respectively (Figure 3C,D).

As a whole, these data suggest that the HFD and DDE treatments reduce mitochondrial tissue content.

### 3.3. Effect of DDE and/or HFD Treatments on Mitochondrial Dynamics

**Figure 4:**
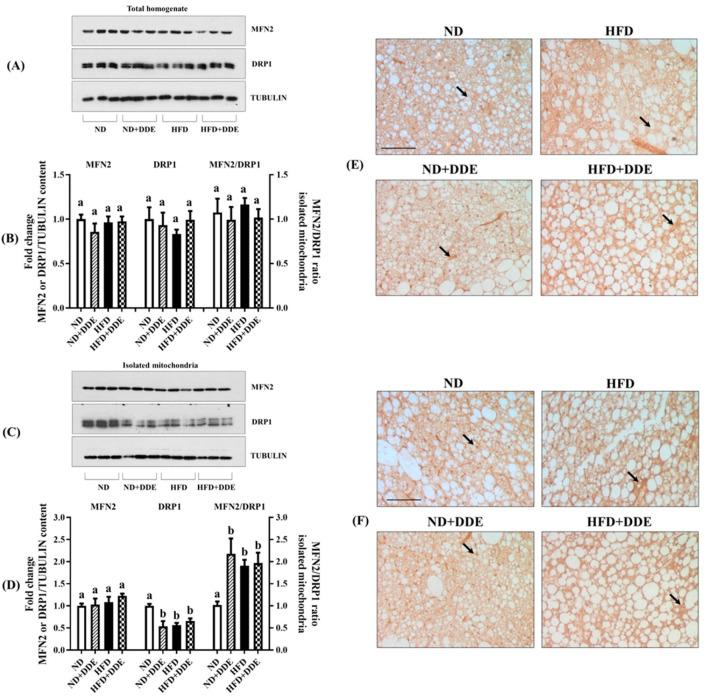
Effect of DDE and/or HFD on mitochondrial dynamics. (**A**) Representative Western Blot of MFN2 and DRP1 and their ratio detected in BAT lysate. (**B**) Histograms represent the quantification of Western blot data. (**C**) Representative Western Blot of MFN2 and DRP1 and their ratio detected in BAT isolated mitochondria. (**D**) Histograms represent the quantification of Western blot data. Tubulin has been used as a loading control. Data were normalized to the values obtained for the ND group, set as 1, and were graphically represented as mean ± SEM of 6 animals per group. Two-way ANOVA analysis, followed by Tukey’s post-test were applied. Bars labelled with different letters are significantly different (*p* < 0.05). (**E**) MFN2 and (**F**) DRP1 immunolocalization. Black arrows indicate immunoreactive evidence of MFN2 and DRP1 localization in the cytosol of brown adipocytes. Magnification used: 20×. Scale bar applied (in ND): 50 μm. Two-way ANOVA analysis: BAT lysates MFN2 levels: effect of diet not significant (n.s.) *p* = 0.5726; effect of DDE n.s. *p* = 0.3592; interaction n.s. *p* = 0.2725. BAT lysates DRP1 levels: effect of diet n.s. *p* = 0.9103; effect of DDE n.s. *p* = 0.5189; interaction n.s. *p* = 0.2211. MFN2/DRP1 ratio detected in BAT lysate: effect of diet n.s. *p* = 0.9488; effect of DDE: *p* = 0.2093; interaction n.s. *p* = 0.6270. Isolated mitochondria MFN2 levels: effect of diet n.s. *p* = 0.1725; effect of DDE n.s. *p* = 0.3849; interaction n.s. *p* = 0.5635. Isolated mitochondria DRP1 levels: effect of diet *p* = 0.0404; effect of DDE *p* = 0.0186; interaction *p* = 0.0010. MFN2/DRP1 ratio detected in isolated mitochondria: effect of diet n.s. *p* = 0.1451; effect of DDE *p* = 0.0134; interaction n.s. *p* = 0.0235.

To further investigate the adaptation occurring in BAT mitochondria following HFD and DDE treatments, we also analyzed the levels of two proteins involved in mitochondrial dynamic behavior, such as mitofusin-2 (MFN2) and dynamic-related protein-1 (DRP1), which are involved in mitochondrial outer membrane fusion and organelles fragmentation, respectively. In the total tissue lysates, no changes in MFN2 and DRP1 levels were observed between the groups (Figure 4A); these data were confirmed by histochemical analyses (Figure 4E,F).

When detected in isolated mitochondria, the DRP1 protein content was significantly reduced by 43%, 34%, and 46% in the HFD, HFD+DDE, and ND+DDE groups vs. the ND group. At the same time, mitochondrial MFN2 content was unchanged between the groups (Figure 4C,D).

We then evaluated the ratio between mitochondrial MFN2 and DRP1, which reflects the organelle fusion and fission balance process, and that was significantly increased by about 1.9 and 2-fold in the HFD and HFD+DDE groups vs. the ND group, and 2,2-fold in the ND+DDE group vs. the ND group, thus suggesting a shift in the mitochondrial dynamic machinery toward fusion.

### 3.4. DDE and HFD Influence on Tissue Oxidative Stress

To gain an understanding about the ability of HFD and DDE to induce oxidative stress in BAT, we detected the total tissue free radicals and TBAR levels associated with SOD2 protein levels, given its role in mitochondrial enzymatic antioxidant defenses.

**Figure 5:**
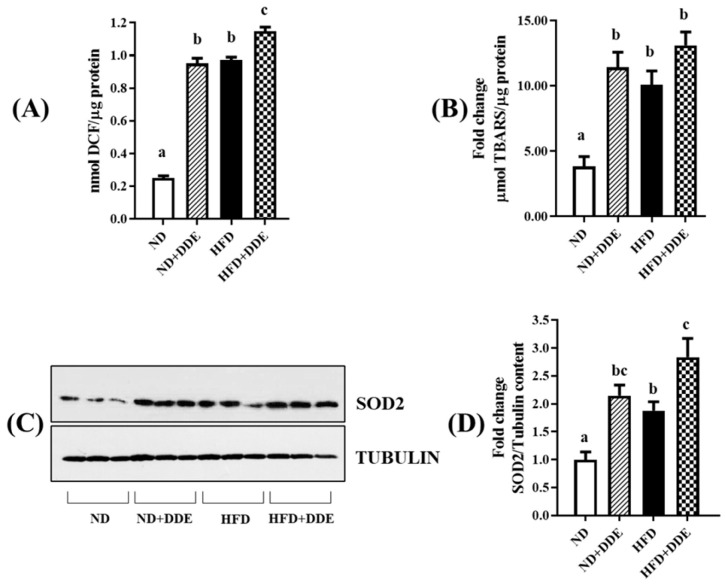
Effect of DDE and/or HFD treatments on oxidative stress parameters detected in BAT. (**A**) Total ROS and (**B**) TBARS levels in total BAT homogenates. (**C**) Representative Western blot of mitochondrial SOD2. (**D**) Histograms represent the quantification of Western blot data. Tubulin has been used as a loading control. For statistical analysis, two-way ANOVA analysis, followed by Tukey’s post-test were applied. Bars labelled with different letters are significantly different (*p* < 0.05). Statistical narrative results evidenced the following effects: ROS levels detected in BAT lysate: effect of diet (*p* < 0.0001); effect of DDE (*p* < 0.0001); interaction (*p* < 0.0001). TBARS levels in BAT lysate: effect of diet (*p* = 0.0008); effect of DDE (*p* < 0.0001); interaction (*p* = 0.0326). SOD2 levels detected in isolated mitochondria: effect of diet (*p* = 0.0020); effect of DDE (*p* = 0.0001); the interaction n.s. (*p* = 0.6733).

As reported in Figure 5, both the HFD and DDE treatments induced BAT oxidative stress. Indeed, the free radical levels detected in the BAT from the HFD, HFD+DDE and ND+DDE groups were 3.8-fold, 4.6-fold, and 3.8-fold higher the ones measured in the ND group, respectively (Figure 5A). Similarly, the HFD and DDE treatments produced an increase in the oxidative damage to lipids. The TBARS levels were increased 2.6-fold, 3.4-fold, and 3.0-fold in the HFD, HFD+DDE, and ND+DDE groups vs. the ND group, respectively (Figure 5B). In line with these results, both the HFD and DDE treatments significantly enhanced the SOD2 protein levels, with the values being increased 1.9-fold, 2.8-fold, and 2.1-fold in the HFD, HFD+DDE, and ND+DDE groups vs. the ND group (Figure 5C,D).

## 4. Discussion

BAT participates in several physiological mechanisms in mammalian species, including thermogenesis and energy balance. Furthermore, recent literature has highlighted the key role of BAT in the development of metabolic diseases [7]. Among the factors capable of influencing the functionality of BAT, chronic exposure to HFDs and environmental pollutants could be included.

The persistent exposure to the environmental contaminant DDE is still a significant public health concern because of its permanency in the environment and in terms of bioaccumulation. In the present paper, we reported novel data concerning the metabolic adaptation observed in BAT following chronic exposure to DDE and concurrent exposure to a HFD. Our data shed light on the ability of DDE to reduce BAT thermogenesis and mimic some of the effects induced by the HFD regime. They also highlight some different BAT responses to chronic exposure to DDE based on the type of diet administered, namely a normolipidic or hyperlipidic diet. As far as we know, it is the first time that the effects of simultaneous exposure to a HFD and DDE have been analyzed in terms of their impact on BAT. Moreover, even if other works have addressed the effect of DDE on BAT [24,30], no deep analysis on the mitochondrial parameters both in tissue lysate and isolated mitochondria have been performed so far.

Concerning chronic HFD feeding, the data reported in the present paper are in line with and confirm previous findings showing that it leads to BAT metabolic impairment [12,13,15]. Indeed, the histological analyses of BAT from rats fed with a HFD show an increase in lipid droplet size that is indicative of less active tissue in terms of thermogenesis. Our data are also in line with the known reduced BAT thermogenesis induced by a HFD and shed light on the similar effect induced by DDE. This is plausibly the result of a decrease in the tissue maximal oxidative capacity, as revealed by the lowered tissue cytochrome oxidase activity. This effect is mainly attributable to the lower mitochondrial tissue content, as can be revealed by the reduction in tissue VDAC/mitochondrial VDAC and tissue UCP1/mitochondrial UCP1 ratios, as well as in PGC1-α protein levels, a transcriptional coactivator that plays a key role in mitochondrial biogenesis and BAT thermogenesis [43]. The PGC1-α protein downregulation induced by DDE is in line with data reported in the literature concerning the ability of the pesticide to reduce PGC1-α mRNA in BAT [23]. In addition, similarly to what was observed in HFD-fed rats, mitochondria isolated from DDE-treated animals, despite being reduced in quantity, were also more active than controls, since the treatments induced an increase in specific cytochrome oxidase activity and UCP1 protein levels.

Another factor that could contribute to the impairment of BAT thermogenesis is the reduction in mitochondrial fragmentation, which, by making mitochondrial bioenergetic processes more efficient, reduces heat production [44,45]. As reported in the present paper, DDE produces a mitochondrial network reorganization toward fusion, as revealed by the reduced mitochondrial DRP1/mitofusin ratio, by mimicking the effect of a HFD. Indeed, our data indicate that in BAT tissue lysates, the levels of proteins involved in mitochondrial dynamics such as MFN2, involved in mitochondrial outer membrane fusion, and DRP1, involved in mitochondrial fission [46], were not affected by the treatments. However, when analyzing isolated mitochondria, DDE and a HFD induced a similar significant decrease in the DRP1 fission protein, without observing changes in MFN2 levels. These results suggest an impaired translocation of DRP1 from the cytosol to mitochondria, and the impossibility of this protein promoting mitochondria fragmentation [47]. Mechanistically, both BAT Drp1 phosphorylation and its translocation to the mitochondria, depend on noradrenaline-induced Protein Kinase-A (PKA) activation [45]. Our data, indicating an impaired translocation of Drp1 to mitochondria, are in line with the ability of DDE to alter the sympathetic regulation of BAT, as reported by Vonderembse et al. [24].

It is known that increased mitochondrial maximal oxidative capacity (evident by the enhanced mitochondrial cytochrome oxidase activity) and improved mitochondrial efficiency (due to the impairment of mitochondria fragmentation) represent two conditions leading to ROS production [48]. This also seems to occur in samples from both DDE and HFD-treated rats in which we observed increased levels of ROS and oxidative damage to lipids. At the same time, some adaptive mechanisms capable of limiting oxidative stress seem to be activated, such as the upregulation of the mitochondrial SOD2 levels, which removes the superoxide anions formed by converting them into H_2_O_2_.

Mitochondrial ROS production is strongly influenced by the proton-motive force present across the inner mitochondrial membrane; therefore, the increase in UCP1 protein levels observed in the mitochondrial fraction induced by DDE by reducing the proton-motive force value, could play a role in limiting ROS production [49]. At the same time, the increase in mitochondrial UCP1 could limit the decline in BAT heat production due to the reduced mitochondrial mass and fragmentation.

It should be considered that DDE mimics the effects induced by a HFD on BAT, and only for some parameters, the effects it induces depend on the diet of the animal.

In particular, the administration of DDE to animals fed with a standard diet does not affect the size of lipid droplets, which are protected by the lipase attack by the upregulation of PLIN-1. Thus, DDE combats the release of fatty acids, which in BAT play a crucial role in thermogenesis. The effect of DDE on PLIN1 upregulation could be due to the ability of organochlorines to stimulate the nuclear translocation of transcription factors that can upregulate lipogenic genes, including PLIN1 [50]. When administered to animals fed with a HFD, DDE exacerbates the accumulation of triglycerides in the tissue and increases the lipid droplet size, thus showing a synergic effect with the HFD.

An additional consideration concerns the whitening of brown adipose tissue. The current literature suggests that a chronic obesogenic diet, as well as chronic exposure to environmental pollutants, such as fine particulate matter, promote brown adipocyte whitening in association with metabolic disorders [19,51]. Our data indicate that chronic exposure to DDE induced an increase in some BAT whitening markers, such as adipocyte morphology with increased lipid droplet size, but only when associated with chronic HFD treatment, thus exacerbating the effect of exposure to the HFD only.

However, the decrease in tissue UCP1 content and mitochondrial mass observed in N+DDE-treated rats suggests that DDE per se induces BAT impairment, which predisposes it to whitening under lipid overload conditions. Further studies are needed to evaluate the effect of DDE on markers of BAT–WAT transdifferentiation, under conditions of a normal or high-fat diet regime.

These considerations reinforce the importance of conducting research, such as that reported in the present study, to shed further light on the effects of environmental pollutants in predisposing cells, tissues, and organs to damage that can favor the development of dysmetabolic pathologies if associated with obesogenic diets.

One limitation to our study is that the results reported refer to male rats, and we did not investigate a potential sexual dimorphism response of BAT to DDE administration.

## 5. Conclusions

In conclusion, our data indicate that exposure to DDE even at doses not considered harmful, by influencing the thermogenic capacity of BAT, can create a predisposition to the onset of energy homeostasis and metabolic disorders. It should be considered that the dose of DDE exposure used in our study is higher than that present in the natural environment. In the literature, it is reported that in Sprague-Dawley dames treated with oral exposure to DDE at a dose of 10 mg/kg p,p′-DDE for 5 days, the DDE levels were approximately 29ppb [52]. These values were comparable to those of 25 ppb found in the serum of pregnant women in the United States [53]. Therefore, our study has relevance from a translational point of view and our consideration has also been reported by Lu et al. [35]. In addition, our studies could help to individuate targets to contrast metabolic disease induced by exposure to environmental pollutant alone, or in association with a hyperlipidemic diet.

The present findings suggest some future research directions. In view of the evidence that DDE mimics most of the effects induced by HFD, it will be interesting to individuate the common factor trigging impaired tissue thermogenesis. Both the HFD and DDE treatment induce oxidative stress, which is well-known to produce deleterious effects on tissue thermogenesis. In this context, it has recently been reported that the progressive deterioration of BAT associated with aging is related to oxidative stress. Furthermore, the in vitro treatment of brown adipocytes with H_2_O_2_ leads to a reduction in the thermogenic capacity of the tissue, which can be attenuated through treatment with antioxidants [54]. Based on this evidence, it is possible to speculate that oxidative stress could underlie the ability of DDE and HFD to induce similar metabolic adaptations in BAT, leading to reduced tissue thermogenesis. Further research studies in which DDE alone, or in combination with HFD, is administered alongside antioxidants, will help support or strike out this possibility.

## Data Availability

The original contributions presented in the study are included in the article, further inquiries can be directed to the corresponding authors.
